# Core values of primary health care help in rethinking the health services

**DOI:** 10.1080/02813432.2023.2189806

**Published:** 2023-03-29

**Authors:** Emil L. Sigurdsson

**Affiliations:** Development Centre for primary healthcare in Iceland, Department of Family Medicine, University of Iceland, ReykjavÍk, Iceland

The healthcare systems in many countries are struggling. They fail to provide adequate services. Both hospitals and primary care providers are dealing with heavy workload and are challenged by lack of personnel. More and more information on burnout and long sick leaves among the employees are seen. The work force is under an immense pressure on delivering both high quality medical service as well as trying to keep up with all the tasks that seem to grow more and more each day. The workers are asked to work harder and to run faster.

The situation in primary care is in many places unacceptable. The fundamental problems are mainly twofold. First, there is a lack of general practitioners (GPs) and second, the primary care is confronted with more and new tasks.

In part, this situation is due to the Covid-19 pandemic. However, that explanation only tells less than half of the story. For many years, it has been obvious that we would need more GPs. Many of them are going on pension within the next few years, the population is both getting older, and the number of inhabitants is increasing. Furthermore, many patients suffer from multimorbidity with complex diseases and polypharmacy.

With longer waiting time to the GP (that is if you are one of the lucky ones to have one) the more likely patients are to ending up visiting out of hour service. Both the emergency departments (ED) of the hospitals and the after-hour service provided by the primary health care are not the places for patients with chronic diseases to attend to. Patients with minor or mild illnesses should be taken care of by a well-manned health care services, preferably during daytime. But understandably patients seek help and visit ED or out of hour services. This leads to more efforts being put on meeting patients with short term illnesses both during daytime as well as during out of hours. The heavy cost is that the care of patients with chronic disease and preventive medicine are not adequately resourced. All this leads to an environment that is, to say the least, harmful to the core values of general practice. We risk developing a fragmented and superficial health care service with poor quality. A service level that most GPs and GP residents are not happy to provide. The consequences are bad service, unsatisfied GPs and most importantly disappointed patients.

In Sweden, the Swedish Association of General Practice (SFAM) together with The Swedish District Medical Association (DLF) have published a report on how to reform primary health care system in a way that it can be the best in the world [1]. The report, published in the second issue of the journal AllmänMedicin 2022, is very thorough and with some useful ideas on how to respond to this crisis. The ideology of general practice is the foundation of these ideas. Emphasizing the importance of *continuity of care, comprehensive care and that everyone has an equal access to care*. These are the same core values that Nordic Colleges of General Practice have formulated to a statement of the Core Values and Principles of Nordic General Practice/Family Medicine [2,3].

## References

[1] https://allmanmedicin.sfam.se/p/allmanmedicin/nr-2-2022/r/4/6-7/1919/534445.

[2] Sigurdsson JA, Baum E, Dijkstra R, et al. HE. Editorial: core values and tasks of primary care in changing communities and health care systems. Front Med. 2022;9:841071. 10.3389/fmed.2022.841071 PMC8859095 35198581

[3] Sigurdsson JA, Beich A, Stavdal A. Our core values will endure. Scand J Prim Health Care. 2020;38(4):363–366. 33377430 10.1080/02813432.2020.1842676 PMC7783035

